# Correlations between antimicrobial peptides and spectrophotometric skin color parameters in patients with basal cell carcinoma

**DOI:** 10.1007/s00432-022-04530-z

**Published:** 2022-12-21

**Authors:** Marta Fijałkowska, Mateusz Koziej, Bogusław Antoszewski, Aneta Sitek

**Affiliations:** 1 Department of Plastic, Reconstructive and Aesthetic Surgery, Second Chair of Surgery Medical University of Lodz, Lodz, Poland; 2 Department of Anatomy, Jagiellonian University Medical College, Cracow, Poland; 3 Department of Anthropology, Faculty of Biology and Environmental Protection, University of Lodz, Lodz, Poland

**Keywords:** Basal cell carcinoma, Cathelicidin, Defensin, Spectrophotometry, Erythema

## Abstract

**Background:**

Antimicrobial peptides (AMPs) are active molecules in the human innate immune system, that participate in host defense and regulate the inflammation process. Previous reports have confirmed that antimicrobial peptides play a critical role in carcinogenesis.

**Objective:**

The present study aimed to evaluate the correlations between plasma concentrations of AMPs and spectrophotometric parameters of skin color in patients with basal cell carcinoma and compare the results with those of healthy controls.

**Methods:**

The plasma concentrations of cathelicidin and beta-defensin-2 in 100 patients (50 with skin cancer and 50 healthy control subjects) were measured, and skin color parameters were tested using a DermaSpectrophotometer.

**Results:**

In patients with basal cell carcinoma, the concentrations of cathelicidin and beta-defensin-2 were significantly higher than those in healthy controls. In healthy control patients, when erythema increases, the levels of cathelicidin and beta-defensin-2 also increase. The direction of the relationship is opposite in people with basal cell carcinoma—the concentration of antimicrobial peptides decreases and the level of erythema increases.

**Conclusion:**

A significantly higher level of plasma concentrations of cathelicidin and HBD-2 are correspondent to the presence of basal cell carcinoma. Skin cancer modifies the relationship between intensity of skin erythema and the levels of cathelicidin and HBD-2. This can be related to inadequate immunological response in patients with skin cancers. New direction of research may be pioneered in searching for cytokine or mast cells disorders.

**Supplementary Information:**

The online version contains supplementary material available at 10.1007/s00432-022-04530-z.

## Introduction

Antimicrobial peptides (AMPs) are active molecules of the human innate immune system that participate in host defense and regulate inflammation process (Koczulla et al. 2003). AMPs play a significant role in enhancing skin immunity, mobilizing epithelial surfaces, and offering different inhibitory effects against many pathogens and emergencies (Gallo and Hooper 2012). More than 1200 AMPs of different origins have been described, of which over 20 are found in human skin (Lai and Gallo 2009). Proteins present in the human skin mainly include cathelicidin, beta-defensins, and dermcidin (Kenshi and Richard 2008).

Cathelicidins are an AMPs family with conserved propeptide sequences that have been described in some mammalian species (Koczulla et al. 2003). Protein LL-37 is the only cathelicidin-derived AMP family identified in humans (Koczulla et al. 2003). LL-37 is found in various cell types such as keratinocytes, respiratory epithelial cells, intestinal cells, neutrophils, monocytes, and mast cells. Thus, LL-37 can be found in the skin, trachea, esophagus, stomach, intestine, liver, spleen, bone marrow, and plasma (Koczulla et al. 2003; Takahashi and Yamasaki 2020; Wang et al. 2016). Four main types of β-defensins have been identified: human beta-defensin-1 (HBD-1), human beta-defensin-2 (HBD-2), human beta-defensin-3 (HBD-3), and human beta-defensin-4 (HBD-4). However, only the first three are present in the skin, and are produced mainly by keratinocytes, neutrophils, and mast cells, which are detected in plasma (Pazgier et al. 2006 Jun; Gambichler et al. 2006).

AMPs show abnormal expression or activity in inflammatory skin diseases such as rosacea, psoriasis, and atopic dermatitis (Takahashi and Yamasaki 2020; Reinholz et al. 2012; Hollox et al. 2008; Park et al. 2018; Hata and Gallo 2008). Additionally studies have confirmed that the expression of HBDs and cathelicidin in patients with different neoplasms is altered (Wang et al. 2016; Gambichler et al. 2006; Fijałkowska et al. 2021a; Scola et al. 2012). Fijałkowska et al. compared the blood concentrations of cathelicidin and HBD2 between 49 patients with BCC and 59 as healthy control (Fijałkowska et al. 2021a). The authors described that the levels of cathelicidin and β-defensin-2 in patients with basal cell carcinoma were significantly elevated and that the specificity of these AMPs in detecting of BCC was high (Fijałkowska et al. 2021a). Gambichler et al. described similar results in the group of 22 patients with BCC while performing mRNA expression of beta-defensins (Gambichler et al. 2006). The authors proved that mRNA expression of hBD-2 was significantly increased in patients with BCC as compared to controls (Gambichler et al. 2006).

Currently, one in every three patients diagnosed with cancer has skin cancer (World Health Organization. How common is skin cancer 2016). Skin cancer (SC) is the most common malignancy, and its incidence is increasing worldwide (Fijałkowska et al. 2021b). The increase in SC incidence is probably caused by a combination of skin color, increased exposure to ultraviolet radiation or sunlight, increased outdoor activities, increased contact with different chemical agents, changes in clothing style, increased longevity, ozone depletion, genetics, and in some cases, immune suppression (Fijałkowska et al. 2021b; Sitek et al. 2016; Leiter et al. 2014). Hence, it has been proven that people with a fair complexion are at an increased risk of developing skin cancer, and descriptive methods of characterizing skin color are confusing. Thus, it is worthwhile to measure skin parameters using an objective method such as spectrophotometry (Sitek et al. 2016; Szczepanek et al. 2021; Abdlaty et al. 2021; Fijałkowska et al. 2022). Some positive relations were described between melanin index and the presence of skin cancer, as melanin index is the simplest spectrophotometric predictor of this disease (Fijałkowska et al. 2022). Therefore, it is interesting to check whether relationships exist between skin color and the level of AMPs in patients with basal cell carcinoma (BCC), as to the best of our knowledge such relationship was not previously reported.

The present study aimed to evaluate the correlations between plasma concentrations of AMPs and spectrophotometric parameters of skin color in patients with BCC in comparison to healthy controls.

## Materials and methods

The study group consisted of 100 people of Polish ancestry (50 patients with BCC and 50 as healthy controls) and was examined between 2020 and 2021. All patients were treated at the Department of Plastic, Reconstructive, and Aesthetic Surgery at Medical University. Written consent to participate in the study was obtained from all patients. The study was approved by the Ethics Review Board of the Medical University of Lodz (approval no. RNN/364/18/KE).

The inclusion criteria were as follows: no diseases related to skin pigmentation or inflammation, no exposure to UV radiation (holidays, solarium) in the last 3 months before the test, no use of skin-bronzing cosmetics, age > 40 years, and no malignancies apart from the skin tumor scheduled for surgical excision. The study group included 50 patients who were admitted for surgical excision for skin lesions suspected of cancer. The diagnosis of skin cancer was based on histopathological examinations. The healthy control group included 50 patients who underwent surgery due to other medical conditions, mainly esthetic conditions.

All patients underwent surgery under local anesthesia for a 1-day surgery. On the day of admission before surgery, a 10 ml blood sample was taken from every patient, and spectrophotometric skin measurements were performed. Spectrophotometric skin measurements were performed using DSM II DermaSpectrophotometer (Cortex Technology, Hadsund, Denmark). The following measurements were performed: melanin index (MI), erythema index (EI), and skin color in the CIELab and RGB color space. Skin color measurements were conducted on the medial regions of the right and left arms, and on the right buttock. The measurements were performed in triplicate in each region, each time at a slightly different location, to avoid melanocytic moles and visible discoloration. Included in the statistical analysis was the mean value obtained from six measurements of the arms and the mean value obtained from three measurements of the buttock. The measurements were done, according to spectrophotometry requirements, in body locations which are not exposed to solar radiation to exclude bias. Performing skin measurements in the most hidden localizations gives the guarantee of taking the most objective measurement of real skin parameters.

The collected blood sample was delivered to the Department of Immunology and Allergy where it was centrifuged to obtain plasma. The sample was frozen and stored at − 80 ºC until analysis. After collecting all 100 samples, the plasma was refrozen, and the concentrations of beta-defensin-1, beta-defensin-2, beta-defensin-3, and cathelicidin were measured using an ELISA kit (EIAab Wuhan Science Co., Ltd).

### Statistical analysis

The Box–Cox transformation was used to normalize the data because the skewed distribution of the analyzed data depended on the measured proteins (dependent variables). Multiple regression was used to assess the relationship between the levels of these proteins and independent variables (sex, age of the patients, presence/absence of skin cancer, and spectrophotometric parameters of skin color on the arms and buttocks). The significance of the interaction between the independent variables was tested using a general linear model (GLM). All analyses were performed using the Statistica package. ver. 13. The level of statistical significance was set at *P* < 0.05.

## Results

The overall characteristics of the study sample is presented in Supplementary Data: Table 1. Because the detection rates of HBD-1 (11 cases) and HBD-3 (1 case) were low, these two defensins were excluded from further analysis. The concentration of HBD-2 was identified in 87 patients (49 with BCC and 38 healthy controls), whereas the concentration of cathelicidin was detected in 86 patients (50 with BCC and 36 healthy controls). These two proteins were included in further analyses.

The levels of cathelicidin and HBD-2 were not associated with patients’ age or sex. However, a statistically significant difference was observed between patients with and without skin cancer. In individuals with skin cancer, the levels of both proteins were higher than those in individuals without skin cancer (Table 1).

**Table 1:** Relationship between cathelicidin/HBD-2 and gender, age, and presence of skin cancer

Independent variable	Dependent variable							
	Cathelicidin after Box–Cox transformation				HBD-2 after Box–Cox transformation			
	*b*	*t*	*p*	*R*^2^_corrected_ [%]	*b*	*t*	*p*	*R*^2^_corrected_ [%]
Male vs female	2.2502	1.73	0.0873	10.67	0.0302	0.91	0.3675	17.20
Age	− 0.0468	− 0.82	0.4160		− 0.0027	− 1.86	0.0657	
Skin cancer presence vs control group	4.5478	3.41	**0.0010**		0.1628	4.76	**0.0000**	

*p* values which are bolded are statisically significant

*b* regression coefficient, *t* test t, *p* probability for test t, *R*^*2*^_*corrected*_ adjusted coefficient of determination

In the course of further analyses, we verified whether there was a relationship between the spectrophotometric parameters of skin color, cathelicidin, and HBD-2 in patients, considering the effects of sex, age, and health (presence or absence of skin cancer). Depending on the spectrophotometric variable included in the model, controlling for the influence of sex and age may be statistically significant or not statistically significant. In the main effects analysis, none of the parameters characterizing the skin color was significantly related to the level of the analyzed proteins (Supplementary data: Table 2).

The significance of correlations between individual spectrophotometric parameters of skin color, sex, age, and the presence of skin cancer was assessed. The analysis revealed that the presence of skin cancer modified the relationship between cathelicidin and erythema index (EI) on the skin of the arms. A similar correlation was observed between EI and HBD-2 concentrations in the skin of the arms. Additionally the correlation between coordinate *a* (CIELab system) and cathelicidin and HBD-2 was revealed for the skin of the arms (Table 2). In the study group, these factors and their interactions accounted for 16–17% of the variability in cathelicidin levels and approximately 20% of the variability in HBD-2 levels. The HBD-2 level also correlated with the EI of the buttock skin, depending on the presence of skin cancer. In the study group, these variables, their relationships, age, and sex of the patients revealed more than 23% variability in the level of this protein (Table 2).

**Table 2:** Results of the general linear model assessing the effect of sex, age, and skin cancer on the relationship between cathelicidin/HBD-2 and spectrophotometric skin color parameters on the arms and buttock

A study of skin color	Independent variable (interactions) ARMS	Dependent variable					
		Cathelicidin after Box–Cox transformation			HBD-2 after Box–Cox transformation		
		*F*	*p*	*R*^2^_corrected_ [%]	*F*	*p*	*R*^2^_corrected_ [%]
Spectrophotometric indicators	MI × sex	0.06	0.8148	10.30	1.76	0.1875	20.34
	MI × age	0.84	0.3608		0.77	0.3812	
	MI × skin cancer	0.49	0.4841		0.06	0.8111	
	EI × sex	0.10	0.7568	16.45	0.02	0.8930	22.07
	EI × age	1.41	0.2380		0.76	0.3867	
	EI × skin cancer	5.67	**0.0193**		4.38	**0.0391**	
CIELab	L × sex	0.00	0.9764	11.61	1.81	0.1814	20.03
	L × age	1.13	0.2911		0.48	0.4892	
	L × skin cancer	2.06	0.1544		1.08	0.3023	
	a × sex	1.29	0.2598	17.53	0.11	0.7366	20.86
	a × age	1.54	0.2185		0.17	0.6791	
	a × skin cancer	5.66	**0.0195**		4.07	**0.0467**	
	b × sex	0.12	0.7339	13.63	1.21	0.2740	18.68
	b × age	0.06	0.8093		0.21	0.6466	
	b × skin cancer	3.25	0.0747		1.30	0.2572	
RGB	R × sex	0.00	0.9834	10.37	0.84	0.3615	20.19
	R × age	1.53	0.2199		1.33	0.2520	
	R × skin cancer	0.11	0.7356		0.02	0.8796	
	G × sex	0.02	0.8963	12.41	1.44	0.2325	20.10
	G × age	1.89	0.1727		0.83	0.3647	
	G × skin cancer	1.63	0.2043		0.53	0.4675	
	B × sex	0.11	0.7403	11.86	2.56	0.1130	20.42
	B × age	0.74	0.3907		0.39	0.5358	
	B × skin cancer	2.00	0.1611		0.25	0.6159	

A study of skin color	Independent variable (interactions) BUTTOCK	Dependent variable					
		Cathelicidin after Box–Cox transformation			HBD-2 after Box–Cox transformation		
		*F*	*p*	*R*^2^_corrected_ [%]	*F*	*p*	*R*^2^_corrected_ [%]
Spectrophotometric indicators	MI × sex	0.05	0.8222	6.71	0.00	0.9825	16.72
	MI × age	0.00	0.9884		0.01	0.9124	
	MI × skin cancer	0.05	0.8264		0.75	0.3893	
	EI × sex	0.18	0.6697	10.66	0.05	0.8277	23.73
	EI × age	1.58	0.2118		0.05	0.8224	
	EI × skin cancer	1.05	0.3090		4.17	**0.0439**	
CIELab	L × sex	0.05	0.8170	6.83	0.11	0.7395	16.35
	L × age	0.01	0.9069		0.00	0.9660	
	L × skin cancer	0.17	0.6845		0.50	0.4825	
	a × sex	0.35	0.5550	12.07	0.06	0.8105	21.34
	a × age	1.82	0.1804		0.56	0.4577	
	a × skin cancer	2.16	0.1450		1.57	0.2135	
	b × sex	0.31	0.5801	9.68	1.54	0.2183	20.63
	b × age	1.07	0.3035		3.16	0.0786	
	b × skin cancer	2.05	0.1560		2.55	0.1136	
RGB	R × sex	0.20	0.6586	8.38	0.14	0.7129	18.70
	R × age	0.00	0.9704		0.03	0.8595	
	R × skin cancer	1.27	0.2619		2.60	0.1100	
	G × sex	0.07	0.7933	6.76	0.11	0.7404	16.31
	G × age	0.10	0.7491		0.03	0.8531	
	G × skin cancer	0.06	0.8011		0.34	0.5606	
	B × sex	0.09	0.7635	6.62	0.02	0.8753	16.12
	B × age	0.01	0.9196		0.02	0.8847	
	B × skin cancer	0.01	0.9381		0.15	0.6958	

*p* values which are bolded are statisically significant

*F* test F, *p* probability for test F, *R*^*2*^_*corrected*_ adjusted coefficient of determination

**Fig. 1:**
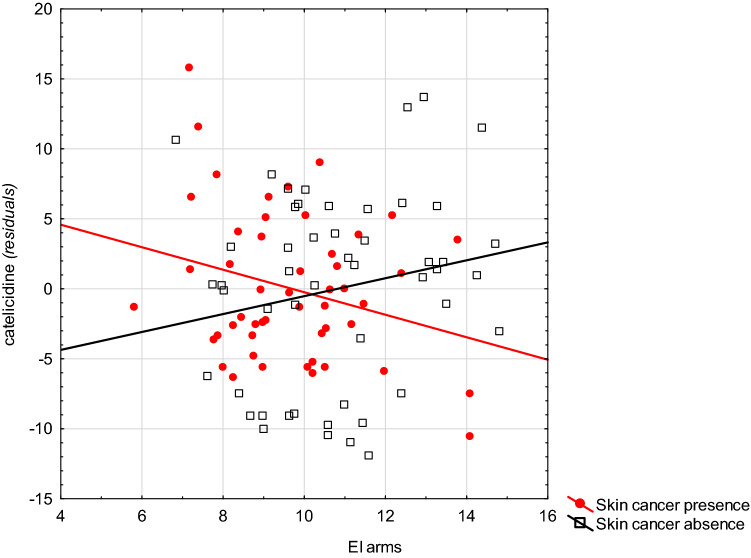
The relationship between the level of erythema of the skin on the arms and the level of cathelicidin in patients with and without skin cancer (Note: residua—residues from the GLM model including the variables explaining gender, age, skin cancer presence, EI arms, and interactions: gender × EI arms, age × EI arms)

**Fig. 2:**
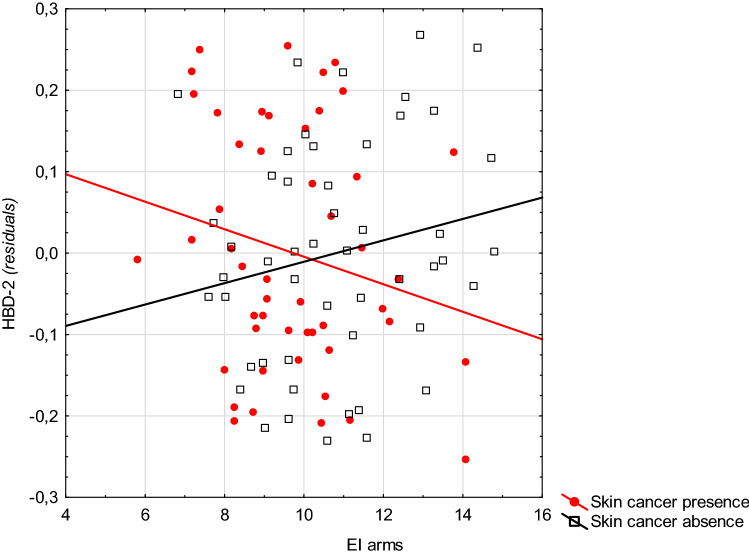
Relationship between the level of erythema of the skin on the arms and the level of HDB-2 in patients with and without skin cancer (Note: residua—residues from the GLM model including the variables explaining gender, age, skin cancer presence, EI arms, and interactions: gender × EI arms, age × EI arms)

**Fig. 3:**
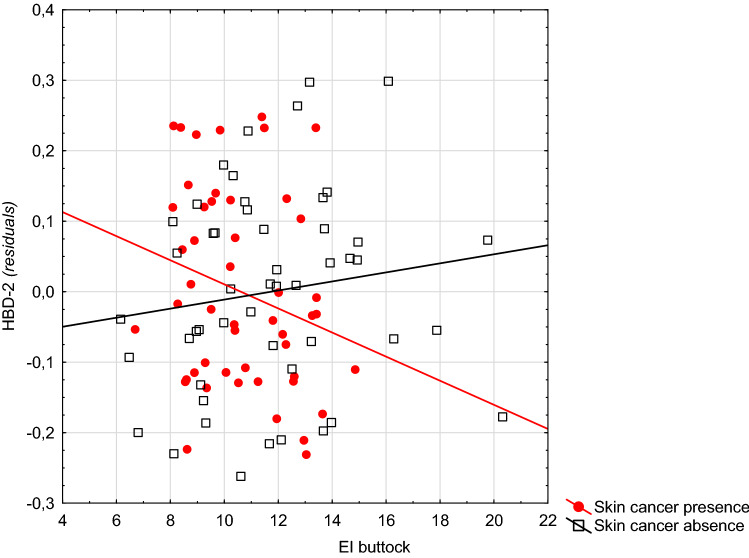
The relationship between the level of erythema of the skin on the right buttock and the level of HDB-2 in patients with and without skin cancer (Note: residua—residues from the GLM model including the variables explaining gender, age, group membership, EI buttock, and interactions: gender × EI buttock, age × EI buttock)

In patients with skin cancer, the graphic presentation of these interactions indicates that a lower EI or *a* coordinate (intensity of red) of the skin of the arms is associated with higher levels of cathelicidin than those without skin cancer. This situation was reversed with higher values of the spectrophotometric parameters (Fig. 1, Supplementary Fig. 1). A similar situation occurred in the relationship between the EI of the skin of the arms and buttock and the HBD-2 level (Fig. 2, Supplementary Fig. 2). A similar situation also occurred between parameter *a* (CIELab) of the skin of the arms and the level of this protein (Fig. 3).

## Discussion

Since skin neoplasms represent an important health problem and the cause of economic burden on society and health services, it is vital to find models or markers to identify patients at increased risk of developing skin cancer, prevent tumor occurrence, and diagnose neoplasms at a very early stage. Such models should be based on markers that can be verified easily. For example, blood samples offer reliable measurements, that are easy to perform and repeat, but are invasive. As non-invasive method, also easy to perform, is spectrophotometric measurements, which offer a suitable, objective, and reproducible method for evaluating pigmentation and skin color (Taylor et al. 2006).

AMPs have a broad spectrum of activities including carcinogenesis as one of them. Shi et al. discovered that upregulation of HBD-2 stimulates growth and invasion during esophageal carcinogenesis. Arimura et al. argued that AMP levels were increased in patients with lung cancer. Wang et al. also investigated the role of cathelicidin in skin squamous cell carcinoma (Wang et al. 2016; Shi et al. 2014; Arimura et al. 2004). In previous studies, it was revealed that plasma levels of LL-37 and HBD-2 were significantly elevated in patients with basal cell carcinoma compared with healthy controls (Fijałkowska et al. 2021a). This association was confirmed in the current study, supporting the theory that these proteins are important molecules playing a role in genesis of skin cancer.

LL-37 activate mast cells to chemotaxis, degranulation, and releasing the pro-inflammatory cytokines, what induce skin inflammation and erythema (Choi et al. 2019). Moreover, Muto et al. demonstrated that mast cells-deficient mice did not develop erythema features after cathelicidin injection (Muto et al. 2014). In our study, it was confirmed that, in healthy controls, the higher the concentration of cathelicidin the bigger erythema, as the stimulation of mast cells grows proportionally to the level of LL-37. The opposite situation was observed in patients with BCC what suggest some immunological deficiencies like insufficient level of mast cells or their lack of ability to degranulate. Studies mentioned above and our results not only emphasize the importance of mast cells in cathelicidin-induced erythema but also provide new direction of potential research among skin cancer patients.

HBD-2 is also capable of potentiating immune responses (Cieślik et al. 2021). There are studies describing a link between HBD-2 expression and production of IL-4, IL-13, IL-17, and IL-18 (Cieślik et al. 2021; Lee et al. 2016; Kolbinger et al. 2017). Some of these interleukins are important cytokines during inflammation what also suggest that the higher the level of defensin-2 the bigger erythema. Opposite direction of relation shown in our patients with BCC may be the result of inadequate cytokine storm or some errors during cytokine cascade what prevents proper immunological defense.

This study had some limitations. First, only a limited number of samples was used. Thus, future research is needed to prove correlations between AMPs and skin color in patients with basal cell carcinoma, and it is worth examining such relationships in patients with other types of skin neoplasms, such as squamous skin carcinoma or melanoma. Nevertheless, the strength of our study is that such relationships have not been previously examined, and to our best knowledge, this is the first report describing it.

## Conclusion

A significantly higher level of plasma concentrations of cathelicidin and HBD-2 is correspondent to the presence of basal cell carcinoma. Skin cancer modifies the relationship between intensity of skin erythema and the levels of cathelicidin and HBD-2. This can be related to inadequate immunological response in patients with skin cancers. New direction of research may be pioneered in searching for cytokine or mast cells disorders in patients who are at increased risk of developing skin cancer. Since there is no single factor responsible for skin cancer development, it is important to choose the most significant ones, especially those that are easy to measure, objective, and reliable. Taking blood samples and performing spectrophotometric measurements seems to be one of them and can be in future markers which allow to identify patients predisposed to skin cancer.

## Supplementary Information

Below is the link to the electronic supplementary material.

Supplementary file1 (DOCX 59 KB)

## Data Availability

All relevant data are within the paper and its supporting information files.
