# Supplementary material for: Correlations between antimicrobial peptides and spectrophotometric skin color parameters in patients with basal cell carcinoma
Source: J Cancer Res Clin Oncol. 2022 Dec 21;149(9):5697–704. doi: 10.1007/s00432-022-04530-z (PMC10356866; doi:10.1007/s00432-022-04530-z)
Supplement: Supplementary file 1 — Supplementary file1 (DOCX 59 KB) [file 432_2022_4530_MOESM1_ESM.docx]

Supplementary Data: Tab. 1 Characteristics of the study group

| Variable | | N=100 (100%) | | | | | | | |
| --- | --- | --- | --- | --- | --- | --- | --- | --- | --- |
|  |  | n | ‾X | SD | Me | Q_1_ | Q_3_ | min | max |
| Sex M/F | | 49/51 |  |  |  |  |  |  |  |
| Skin cancer group/Skin cancer absence | | 50/50 |  |  |  |  |  |  |  |
| Age | | 100 | 66.10 | 11.71 | 67.00 | 56.00 | 75.00 | 43.00 | 90.0 |
| Cathelicidin | | 100 | 705.22 | 1146.66 | 351.87 | 112.04 | 705.80 | 0.00 | 6778.9 |
| HBD-2 | | 100 | 0.88 | 1.47 | 0.33 | 0.15 | 0.63 | 0.00 | 6.7 |
| ARMS | | | | | | | | | |
| Spectrophotometric indicators | MI | 100 | 28.36 | 3.05 | 28.35 | 25.93 | 29.62 | 21.21 | 38.2 |
|  | EI | 100 | 10.26 | 1.98 | 10.07 | 8.86 | 11.42 | 5.82 | 14.8 |
| CIELab | L | 100 | 44.17 | 4.01 | 44.61 | 41.30 | 47.18 | 35.39 | 51.2 |
|  | a | 100 | 14.82 | 2.65 | 14.39 | 13.21 | 16.48 | 9.74 | 22.4 |
|  | b | 100 | 14.43 | 2.67 | 14.12 | 12.23 | 16.13 | 9.24 | 22.3 |
| RGB | R | 100 | 131.92 | 9.55 | 132.08 | 125.50 | 137.83 | 105.67 | 156.5 |
|  | G | 100 | 105.37 | 10.60 | 106.33 | 97.83 | 111.58 | 82.17 | 126.0 |
|  | B | 100 | 102.07 | 12.19 | 102.75 | 94.08 | 109.83 | 76.83 | 128.7 |
| BUTTOCK | | | | | | | | | |
| Spectrophotometric indicators | MI | 100 | 26.86 | 4.05 | 26.56 | 23.77 | 29.47 | 20.44 | 39.8 |
|  | EI | 100 | 11.16 | 2.63 | 10.78 | 9.18 | 12.78 | 6.16 | 20.3 |
| CIELab | L | 100 | 44.37 | 5.16 | 44.62 | 41.08 | 48.37 | 28.79 | 55.5 |
|  | a | 100 | 16.67 | 3.74 | 16.44 | 14.27 | 19.27 | 9.15 | 27.8 |
|  | b | 100 | 13.55 | 3.79 | 13.89 | 10.90 | 16.23 | 3.14 | 22.7 |
| RGB | R | 100 | 136.34 | 12.06 | 137.00 | 128.17 | 145.83 | 102.00 | 159.3 |
|  | G | 100 | 105.88 | 13.64 | 107.00 | 96.67 | 116.50 | 64.67 | 138.3 |
|  | B | 100 | 103.91 | 14.18 | 106.00 | 92.67 | 112.17 | 57.00 | 132.3 |

n- number, x- mean, SD -standard deviation, Me-median, Q_1_ – lower quartile, Q_3_ – upper quartile, MI – melanin index, EI- erythema index, CIELAB - color system, RGB - color system,

Supplementary data: Tab. 2. Multiple regression results assessing the relationship between cathelicidin/HBD-2 and spectrophotometric parameters of skin color on the arms and buttocks

| A study of skin color | Independent variable* | Dependend variable | | | | | | | |
| --- | --- | --- | --- | --- | --- | --- | --- | --- | --- |
|  |  | Cathelicidin  after Box-Cox transformation | | | | HBD-2  after Box-Cox transformation | | | |
|  |  | b | t | p | R^2^_corrected._  [%] | b | t | p | R^2^_corrected._  [%] |
| ARMS | | | | | | | | | |
| Spectrophotometric indicators | MI | 0.2804 | 1.30 | 0.1977 | 11.00 | 0.0057 | 1.04 | 0.3010 | 17.27 |
|  | EI | 0.1899 | 0.52 | 0.6014 | 9.68 | -0.0012 | -0.13 | 0.8935 | 16.35 |
| CIELab | L | -0.1116 | -0.66 | 0.5127 | 9.83 | -0.0043 | -1.01 | 0.3149 | 17.22 |
|  | a | -0.3289 | -1.12 | 0.2633 | 10.61 | -0.0058 | -0.78 | 0.4357 | 16.87 |
|  | b | 0.2960 | 1.17 | 0.2415 | 10.73 | 0.0057 | 0.89 | 0.3772 | 16.28 |
| RGB | R | -0.090 | -1.33 | 0.1845 | 11.09 | -0.0024 | -1.56 | 0.1210 | 20.54 |
|  | G | -0.049 | -0.77 | 0.4421 | 9.99 | -0.0018 | -1.15 | 0.2516 | 17.49 |
|  | B | -0.058 | -1.07 | 0.2866 | 10.50 | -0.0015 | -1.08 | 0.2807 | 17.35 |
| BUTTOCK | | | | | | | | | |
| Spectrophotometric indicators | MI | 0.064 | 0.38 | 0.6983 | 9.57 | 0.0024 | 0.59 | 0.5573 | 16.63 |
|  | EI | -0.124 | -0.44 | 0.6546 | 9.61 | -0.0113 | -1.61 | 0.1101 | 18.56 |
| CIELab | L | -0.044 | -0.33 | 0.7366 | 9.53 | -0.0006 | -0.20 | 0.8399 | 16.37 |
|  | a | -0.209 | -1.05 | 0.2935 | 10.47 | -0.0078 | -1.55 | 0.1249 | 18.39 |
|  | b | -0.167 | -0.97 | 0.3304 | 10.33 | -0.0038 | -0.88 | 0.3798 | 17.01 |
| RGB | R | -0.036 | -0.67 | 0.5011 | 9.86 | -0.0010 | -0.75 | 0.4571 | 16.82 |
|  | G | -0.001 | -0.02 | 0.9796 | 9.42 | 0.0000 | 0.05 | 0.9586 | 16.33 |
|  | B | 0.006 | 0.13 | 0.8959 | 9.44 | 0.0002 | 0.19 | 0.8488 | 16.36 |

* adjusted for gender, age and skin cancer presence

b- regression coefficient, t- test t, p- probability for test t, R^2^_corrected_ – adjusted coefficient of determination

Supplementary Fig 1. The relationship between the coordinator a (CIELab) for the skin on the arms and the level of cathelicidin in patients with and without skin cancer (Note: residua - residues from the GLM model including the variables explaining gender, age, skin cancer presence, a arms and interactions: gender x a arms, age x a arms)

Supplementary Fig 2. The relationship between the coordinator a (CIELab) for the skin on the arms and the level of defensin 2 in patients with and without skin cancer (Note: residua - residues from the GLM model including the variables explaining gender, age, skin cancer presence, a arms and interactions: gender x a arms, age x a arms)
